# 348. Characteristics and Outcomes in Hospitalized Patients with Covid-19 Complicated by Fungemia: A Single Center Retrospective Study

**DOI:** 10.1093/ofid/ofab466.549

**Published:** 2021-12-04

**Authors:** Aleena Zahra, Bettina C Fries, Bettina C Fries, Sutthichai Sae-Tia

**Affiliations:** 1 SUNY Stony Brook University Hospital, Stony Brook, New York; 2 Stony Brook University Hospital, Stony Brook, NY

## Abstract

**Background:**

Covid19 caused by SARS-CoV2 can lead to significant morbidity and mortality. Fungemia is a rare hospital-associated infection and there are limited data regarding its association with Covid19. We reviewed all cases of fungemia in our Covid19 cohort at Stony Brook University Hospital (SBUH).

**Methods:**

We conducted a retrospective medical record review of patients admitted with Covid19 in a 3-month interval. We reviewed positive blood cultures for fungi and recorded co-morbidities, co-infections, length of stay, treatments, and outcomes (survival vs death). There were 60 positive blood cultures for fungi in 25 unique patients (Table 1); in prior years < 30 per year reported at SBUH.

Table 1. Fungal Blood Cultures

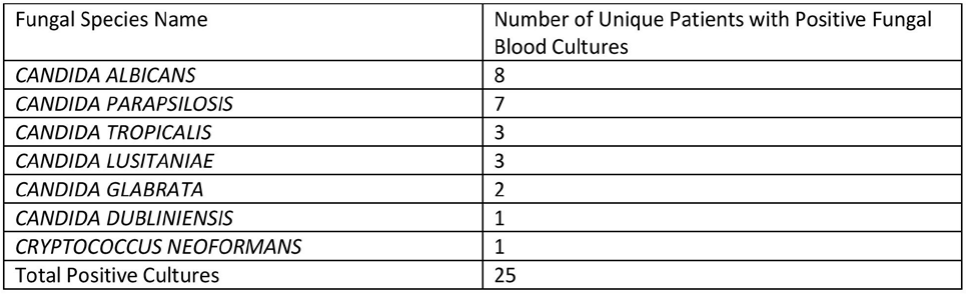

Collation of each unique identified fungal species from fungal blood cultures in patients hospitalized with Covid-19

**Results:**

During a 3 month interval at the local peak of the pandemic 1398 patients hospitalized with Covid19 at SBUH, 25 cases of fungemia were detected; *C. albicans* (CA) n=8,32%, non *C albicans* species (nCA) n=16,64%, and *C. neoformans* n=1,4%, 17/25 (68%) also with bacteremia during same hospitalization. In same 3 months there were 264 cases of bacteremia and Covid19 co-infection. Demographics and medical co-morbidities of fungemic patients are in Table 2. Majority were men (76%). No difference between fungaemic (FC) and total cohort (TC) in median age (62 vs 62), DM p=0.31, HTN p=1.0, COPD p=0.12. Within FC, DM was higher in nCA group (58.8%) vs CA group (37%). Mortality was 40% in FC vs 15% in TC, p< 0.001. Within FC mortality was 56% in nCA and 25% in CA group. *C. parapsilosis* was the most common nCA species isolated with 43% mortality. FC more likely to require ICU and mechanical ventilation (88% vs 15%, p< 0.0001) and had longer median length of stay 42 days vs 22 days. The median time from admission to fungaemia was 21d, from central line placement 19d, Table 3. Of FC 21 (84%) were treated with steroids/Tocilizumab concurrently. Of note, no mortality was recorded in the 4 patients that did not receive steroids/Tocilizumab.

PCT and WBC were significantly higher at time of fungemia as compared to admission, Table 3.

Table 2, Patient co-morbidities and hospitalization stay characteristics

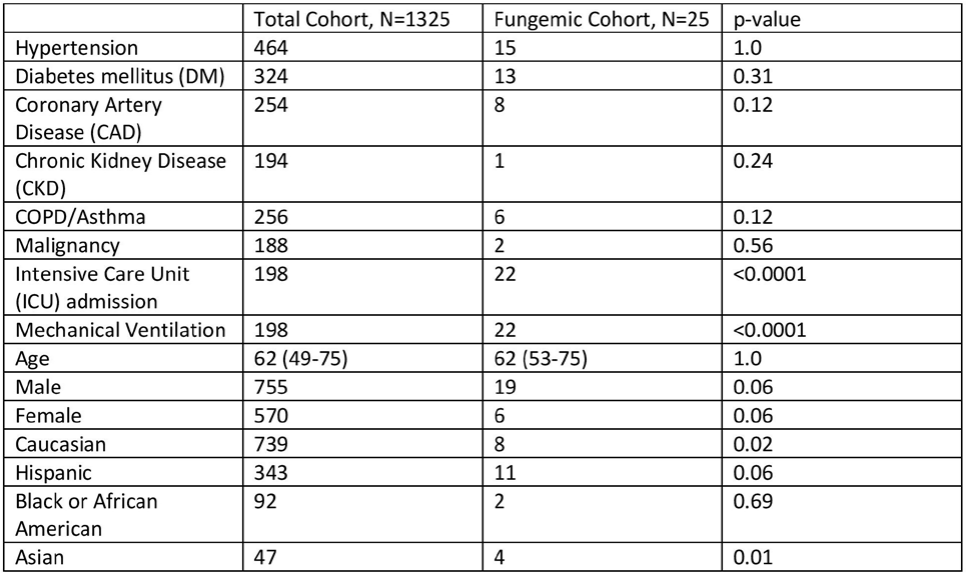

Co-morbidities and requirement for ICU stay, mechanical ventilation for total cohort Covid-19 and fungemic cohort

Table 3, Patient Characteristics and Laboratory Parameters

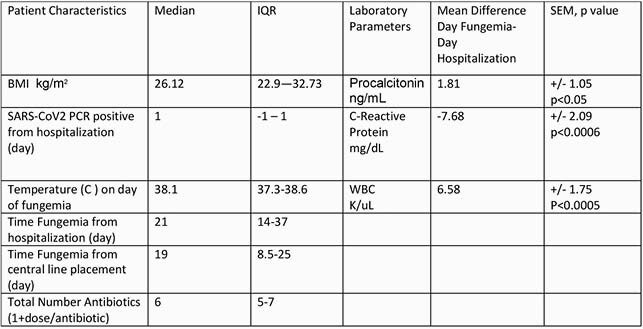

Relevant patient characteristics and laboratory parameters in patients hospitalized with Covid19 and fungemia

**Conclusion:**

Fungemia in hospitalized patients with COVID-19 is associated with higher mortality. We observed higher fatality in non *C. albicans* infections. Prolonged use of central line catheters and concurrent treatment with steroids/tociluzimab are likely high-risk factors for development of fungemia.

**Disclosures:**

**All Authors**: No reported disclosures

